# Supplementary material for: Matching sensor ontologies through siamese neural networks without using reference alignment
Source: PeerJ Comput Sci. 2021 Jun 18;7:e602. doi: 10.7717/peerj-cs.602 (PMC8237319; doi:10.7717/peerj-cs.602)
Supplement: Supplemental Information 1 [file peerj-cs-07-602-s001.zip › 266/onto.html]

Author: Nick Knouf <nknouf@mit.edu>  
Contributor: Antoine Zimmermann <antoine.zimmermann@inrialpes.fr>, Jérôme Euzenat,   
Date: 08/06/2005  
Version: $Id: onto-exp.rdf,v 1.10 2008/05/27 14:40:02 euzenat Exp $

## Classes

**http://www.w3.org/1999/02/22-rdf-syntax-ns#List** (, *)*


**http://xmlns.com/foaf/0.1/Person** (, *)*


**http://xmlns.com/foaf/0.1/Organization** (, *)*


**uqwz** (, *)*
:   **sqdsq** (, *)*
    :   **PBdgcs** (, *)*
        :   **zajdhqs** (, *)*
            :   **dzajndsq** (, *)*


                **sqxsqkd** (, *)*


                **zdqssqdb** (, *)*

            **qsdsnbsqd** (, *)*
            :   **zdzaudbd** (, *)*


                **scds** (, *)*


                **dcsqdsq** (, *)*
                :   **ZikPzsz** (, *)*


                    **ZGKW1E** (, *)*

                **zeadza** (, *)*

            **sqdsopq** (, *)*
            :   **RJbGVM** (, *)*
                :   **hazdn** (, *)*


                    **Be** (, *)*


                    **fGfFm** (, *)*


                    **wFuU** (, *)*

                **dzqndbzq** (, *)*


                **vccfsq** (, *)*


                **dcsqdcsqd** (, *)*


                **deqdxcsqcsq** (, *)*

            **zdazsx** (, *)*
            :   **xsqlknk** (, *)*


                **zqdzqzai** (, *)*

            **wxsdxqsdh** (, *)*


            **dqzdxdcsqj** (, *)*
            :   **EwQu** (, *)*
                :   **xhNJuGU** (, *)*


                    **UWmlX** (, *)*


                    **FPVijs** (, *)*
                    :   **cnNCBO** (, *)*
                        :   **ZVxsNo** (, *)*


                            **jZVxsNo** (, *)*

                        **Lluuk** (, *)*
                        :   **sdcsqhyz** (, *)*


                            **QMgwFB** (, *)*


                            **YeLgZJu** (, *)*


                            **LXtNU** (, *)*

                    **XaWa** (, *)*
                    :   **FkmaDDV** (, *)*

                **azzauhuie** (, *)*
                :   **pjDVyIr** (, *)*

        **dscdscg** (, *)*

**BeXCIG** (, *)*
:   **qsdsquj** (, *)*


    **wxVRZcIXbbz** (, *)*

**cexQ** (, *)*
:   **zqedzbx** (, *)*


    **RcrfZK** (, *)*


    **qHdl** (, *)*


    **izVyrw** (, *)*

**qzddj** (, *)*


**izxnquo** (, *)*
:   super: *http://xmlns.com/foaf/0.1/Organization*  


    **YpNk** (, *)*


    **zauio** (, *)*


    **BriFsw** (, *)*
    :   **MyRFOvC** (, *)*


        **zadazxn** (, *)*


        **SNNu** (, *)*


        **SLoVq** (, *)*

**dsqdbz** (, *)*
:   super: *http://www.w3.org/1999/02/22-rdf-syntax-ns#List*

**dnqsb** (, *)*


**dzshyuua** (, *)*

## Properties

**http://www.w3.org/1999/02/22-rdf-syntax-ns#first**: http://www.w3.org/1999/02/22-rdf-syntax-ns#List -> \_ *()*


**http://www.w3.org/1999/02/22-rdf-syntax-ns#rest**: http://www.w3.org/1999/02/22-rdf-syntax-ns#List -> http://www.w3.org/1999/02/22-rdf-syntax-ns#List *()*

**http://purl.org/dc/elements/1.1/creator**\_ -> \_ *()*


**http://purl.org/dc/elements/1.1/contributor**\_ -> \_ *()*


**http://purl.org/dc/elements/1.1/description**\_ -> \_ *()*


**http://purl.org/dc/elements/1.1/date**\_ -> \_ *()*


**http://xmlns.com/foaf/0.1/firstName**\_ -> \_ *()*


**#lastName**\_ -> \_ *()*


**http://xmlns.com/foaf/0.1/name**\_ -> \_ *()*

## Individuals

<rdf:List@ttp://www.w3.org/1999/02/22-rdf-syntax-ns#nil>

---

Generated by OWL2HTML
